# Headache and Neurological Symptoms Following Lumbar Puncture in a Patient With a Ventriculoperitoneal Shunt

**DOI:** 10.7759/cureus.97293

**Published:** 2025-11-19

**Authors:** Nicholas Koenig, Stephen Howell, Arpan Kohli, Jason Shepherd

**Affiliations:** 1 Anesthesiology, West Virginia University School of Medicine, Morgantown, USA

**Keywords:** epidural blood patch, focal seizures, injection drug use, post-dural puncture headache, ventriculo-peritoneal shunt surgery

## Abstract

Post-dural puncture headache (PDPH) is a common complication of lumbar puncture and neuraxial anesthesia, typically resulting from cerebrospinal fluid (CSF) leakage and intracranial hypotension. Although the pathophysiology and management of PDPH are well established in the general population, its diagnosis and treatment in patients with ventriculoperitoneal shunts pose unique challenges due to altered CSF dynamics and intracranial compliance. In these individuals, even minor CSF volume changes can produce unpredictable pressure gradients and paradoxical phenomena such as low-pressure hydrocephalus. The use of an epidural blood patch, a mainstay of PDPH management, carries additional risks in this population and must be approached with multidisciplinary collaboration and careful hemodynamic consideration.

## Introduction

Post-dural puncture headache (PDPH) is a well-recognized complication of lumbar puncture and neuraxial anesthesia, resulting from cerebrospinal fluid (CSF) loss through a dural defect and subsequent intracranial hypotension. The classic presentation is an orthostatic headache that worsens in the upright position and improves when supine, frequently accompanied by neck stiffness, nausea, and photophobia [1-3]. The underlying pathophysiology involves a combination of downward traction on pain-sensitive intracranial structures, compensatory meningeal vasodilation, and mechanical deformation of the brain and meninges. Although many cases resolve spontaneously, persistent or severe PDPH can be debilitating and often requires definitive treatment with an epidural blood patch. By injecting autologous blood into the epidural space, the epidural blood patch seals the dura and restores normal CSF pressure, with success rates reported between 70% and 90% [4].

However, anesthetic and diagnostic management of PDPH becomes considerably more complex in patients with ventriculoperitoneal (VP) shunts. Shunted patients have altered CSF hydrodynamics and intracranial compliance, predisposing them to exaggerated or unpredictable pressure fluctuations after lumbar puncture or epidural procedures [5]. In these individuals, even modest CSF removal can precipitate profound intracranial hypotension. At the same time, subsequent interventions, such as an epidural blood patch, may transiently elevate intracranial pressure or interfere with shunt drainage [6]. Rarely, such patients may develop low-pressure hydrocephalus, characterized by ventricular dilation despite low measured intracranial pressure, which can paradoxically improve after restoration of CSF pressure through an epidural blood patch [7]. Distinguishing among PDPH, low-pressure hydrocephalus, and true shunt malfunction requires a careful understanding of CSF physiology and a multidisciplinary approach involving anesthesiology, neurosurgery, and radiology.

Procedural and peri-procedural challenges may further complicate management. Individuals with a history of intravenous (IV) drug use often present with significant scarring and venous sclerosis that make vascular access extremely difficult [8]. In such cases, establishing IV access for hydration, sedation, or emergency medications during neuraxial procedures can become a major technical obstacle. During an epidural blood patch, a sterile IV must be placed to draw blood for the procedure, which is often the most challenging part, especially in patients with poor IV access sites. In the present case, difficult IV access due to prior IV drug use prolonged pre-procedural preparation and complicated the administration of supportive therapy during the epidural blood patch, highlighting the importance of pre-procedure planning in similar patient populations.

We describe the case of a 38-year-old woman with a history of a VP shunt who developed severe post-lumbar puncture headache, back pain, and lower-extremity numbness following diagnostic spinal fluid drainage.

## Case presentation

A 38-year-old woman with idiopathic intracranial hypertension and a seizure disorder managed with levetiracetam and lamotrigine and a history of IV drug use presented to the emergency department with severe headache, back pain, and left-leg numbness following a lumbar puncture. She had undergone placement of her first VP shunt in May 2024. She had a remote history of IV drug use that made peripheral venous access challenging, necessitating the use of ultrasound for peripheral venous access. Four days prior to the presentation, she underwent a lumbar puncture to evaluate possible shunt malfunction. Approximately 30 mL of CSF was removed during the procedure, which was performed without immediate complications.

**Figure 1:**
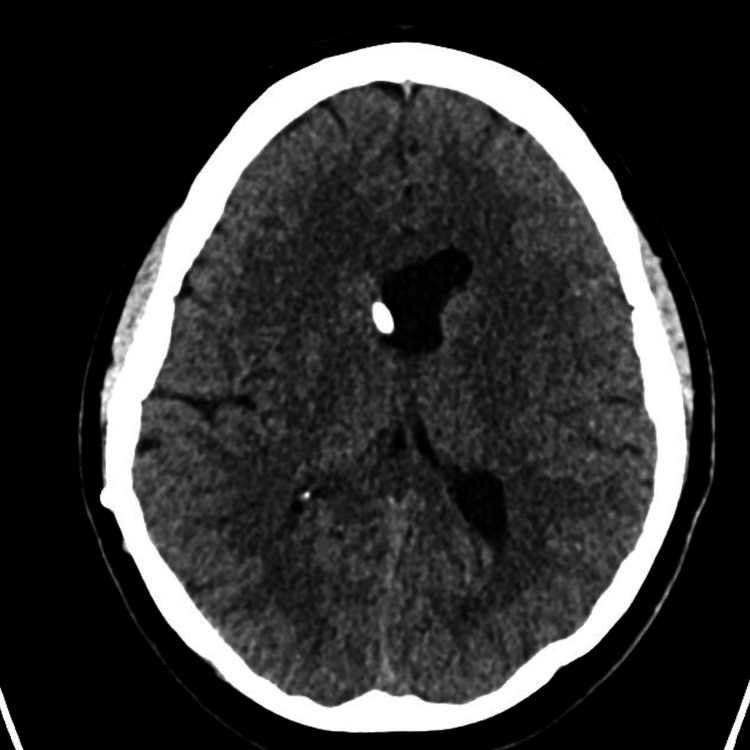
Noncontrast CT brain This figure shows a noncontrast CT of the brain in July 2025, demonstrating the patient's VP shunt in the lateral ventricle. CT: computed tomography, VP: ventriculoperitoneal

**Figure 2:**
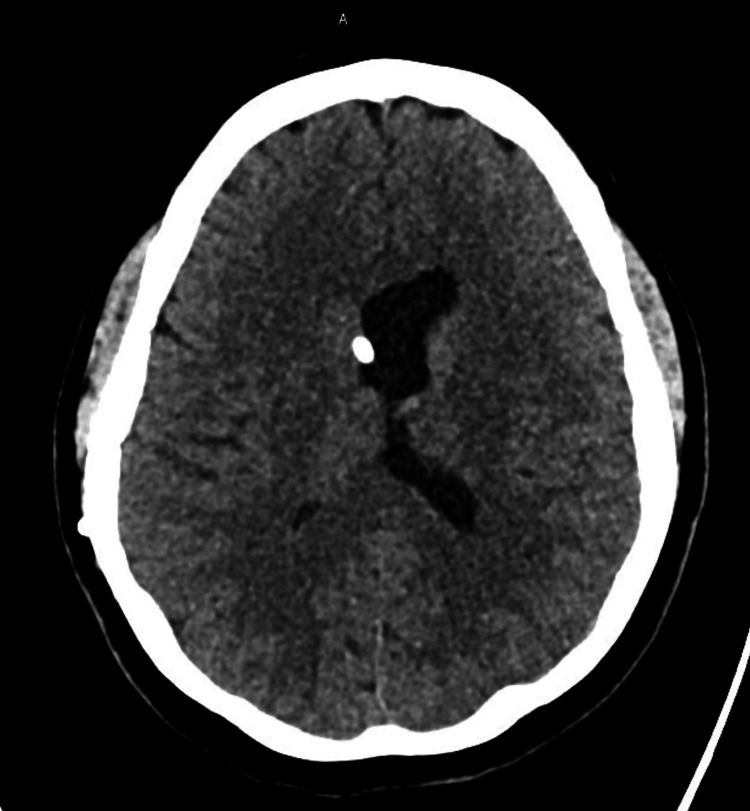
Noncontrast CT brain This figure shows a noncontrast CT of the brain in September 2025, demonstrating the stable position of the patient's VP shunt in the lateral ventricle. CT: computed tomography, VP: ventriculoperitoneal

Within 24 hours, she developed a severe orthostatic headache accompanied by nausea and low-back pain radiating into the lower extremities. She also described numbness in the left leg but denied fever, urinary retention, or bowel changes. Conservative therapy with two liters of IV fluids, oral hydration, caffeine, and analgesia such as ketorolac was ineffective for two days. A CT scan of the brain revealed an unchanged configuration of the lateral ventricles with the VP shunt in place (Figures 1-2). After counseling and informed consent, an epidural blood patch was performed. The procedure was completed without immediate complications, and she experienced rapid and nearly complete resolution of her headache and back pain.

Approximately 24 hours later, she returned with a recurrence of a headache that she described as qualitatively different from the original. The new pain was non-positional, localized to the left side of her head, and radiated toward the frontal region. She also reported worsening lower back pain radiating into the abdomen, persistent paresthesias in both legs (more pronounced on the left), and a small episode of urinary incontinence. During evaluation, she exhibited a brief episode of seizure-like activity witnessed by staff. Neurological examination revealed diminished sensation in the left L5 dermatome and mild dorsiflexion weakness, with symmetric but brisk ankle reflexes. Neurosurgery and anesthesia were consulted for evaluation of possible shunt malfunction, recurrent CSF leakage, or complications related to the epidural blood patch. Prior to a full workup, the patient left against medical advice and did not return to the emergency department within the subsequent days.

## Discussion

This case highlights the diagnostic uncertainty that arises when a patient with a VP shunt develops recurrent headache and new neurological findings after a lumbar puncture and epidural blood patch. The patient’s initial presentation was consistent with PDPH due to CSF leakage, given the orthostatic nature of the pain, temporal association with the lumbar puncture, and rapid resolution after the epidural blood patch. The epidural blood patch likely re-established epidural pressure and sealed the dural defect, transiently restoring normal CSF volume and relieving intracranial hypotension. However, the subsequent recurrence of headache with altered character, non-positional and lateralized, along with the development of leg paresthesias and urinary incontinence, expanded the differential diagnosis to include shunt-related complications, persistent CSF leakage, and procedural complications of the epidural blood patch itself.

In patients with VP shunts, lumbar puncture alters the pressure relationship between the cranial and spinal compartments, occasionally producing paradoxical effects on shunt function. When CSF is drained, a negative pressure gradient may develop, leading to ventricular collapse and reduced shunt flow. This “low-pressure hydrocephalus” has been reported after lumbar puncture and may manifest as severe headache, confusion, or even ventricular enlargement on imaging despite low intracranial pressure [6-7]. Several reports describe resolution of symptoms after epidural blood patch, which re-establishes epidural and subarachnoid equilibrium and restores shunt function. In the present case, the patient’s transient improvement after the epidural blood patch could have resulted from such a mechanism. However, the recurrence of symptoms raises the possibility of ongoing pressure imbalance or shunt dysfunction rather than simple CSF leakage.

Another important consideration is the potential for complications from the epidural blood patch itself. Although the procedure is generally safe, rare events such as epidural hematoma, subdural hematoma, or adhesive arachnoiditis have been documented [9-11]. These complications may present with back pain, radiculopathy, or new motor and sensory deficits. In this case, the emergence of leg paresthesias and urinary incontinence after the epidural blood patch warranted urgent imaging to exclude compressive lesions or inflammatory changes. In addition, difficult vascular access in a patient with prior IV drug use can prolong procedural time and limit the ability to administer sedatives or fluids, increasing patient discomfort and procedural complexity.

From an anesthetic perspective, this case underscores several key principles. First, in shunted patients, the removal of even modest amounts of CSF should be approached with caution and in consultation with neurosurgery, as changes in intracranial compliance can lead to unpredictable pressure dynamics. Second, while an epidural blood patch remains an effective treatment for PDPH, its use in patients with altered CSF flow should be tailored to minimize sudden pressure shifts. Smaller incremental blood volumes, slow injection, and careful neurological monitoring are recommended. Third, any change in headache character or the appearance of new neurological or bladder symptoms after an epidural blood patch should prompt immediate imaging before further neuraxial intervention. Finally, patients with histories of IV drug use often require advanced planning for vascular access, sedation, and post-procedural care, as well as compassionate, stigma-free communication to ensure optimal cooperation and safety.

## Conclusions

This case highlights the complex interplay between CSF physiology, shunt mechanics, and anesthetic intervention. In patients with VP shunts, lumbar puncture and subsequent epidural blood patch require heightened vigilance due to the potential for rapid intracranial pressure shifts, shunt malfunction, or paradoxical low-pressure hydrocephalus. The evolution of symptoms, particularly changes in headache character or the onset of new neurological deficits, should prompt early imaging and multidisciplinary evaluation. Additionally, peri-procedural challenges such as poor vascular access in patients with histories of IV drug use demand preemptive planning and coordination among anesthesia and nursing teams. Ultimately, individualized management and interdepartmental collaboration are key to optimizing outcomes in this uniquely vulnerable population.
